# Uptake of a fluorescently tagged chloroquine analogue is reduced in CQ-resistant compared to CQ-sensitive *Plasmodium falciparum* parasites

**DOI:** 10.1186/s12936-019-2980-y

**Published:** 2019-10-07

**Authors:** Sarah J. Reiling, Petra Rohrbach

**Affiliations:** Institute of Parasitology, McGill University, Ste. Anne de Bellevue, Montreal, QC H9X-3V9 Canada

**Keywords:** LynxTag-CQ_GREEN_, Live cell imaging, Malaria, Antimalarial drugs, Chloroquine resistant, Chloroquine sensitive

## Abstract

**Background:**

Chloroquine (CQ) was the drug of choice for decades in the treatment of falciparum malaria until resistance emerged. CQ is suggested to accumulate in the parasite’s digestive vacuole (DV), where it unfolds its anti-malarial properties. Discrepancies of CQ accumulation in CQ-sensitive (CQS) and CQ-resistant (CQR) strains are thought to play a significant role in drug susceptibility. Analysis of CQ transport and intracellular localization using a fluorescently tagged CQ analogue could provide much needed information to distinguish susceptible from resistant parasite strains. The fluorescently tagged CQ analogue LynxTag-CQ™_GREEN_ (CQ_GREEN_) is commercially available and was assessed for its suitability.

**Methods:**

IC_50_ values were determined for both CQ and CQ_GREEN_ in two CQS and two CQR *Plasmodium falciparum* strains. Buffer solutions with varying pH were used to determine pH-dependent localization of CQ_GREEN_ in infected red blood cells. Before CQS or CQR parasites were exposed to different pH buffers, they were pre-loaded with varying concentrations of CQ_GREEN_ for up to 7 h. Intracellular accumulation was analysed using live cell confocal microscopy. CQ_GREEN_ uptake rates were determined for the cytosol and DV in the presence and absence of verapamil.

**Results:**

In CQS strains, twofold higher IC_50_ values were determined for the CQ_GREEN_ analogue compared to CQ. No significant differences in IC_50_ values were observed in CQR strains. Addition of verapamil reversed drug resistance of CQR strains to both CQ and CQ_GREEN_. Live cell imaging revealed that CQ_GREEN_ fluorescence was mainly seen in the cytosol of most parasites, independent of the concentration used. Incubation periods of up to 7 h did not influence intracellular localization of CQ_GREEN_. Nevertheless, CQ_GREEN_ uptake rates in CQR strains were reduced by 50% compared to CQS strains.

**Conclusion:**

Although fluorescence of CQ_GREEN_ was mainly seen in the cytosol of parasites, IC_50_ assays showed comparable efficacy of CQ_GREEN_ and CQ in parasite killing of CQS and CQR strains. Reduced uptake rates of CQ_GREEN_ in CQR strains compared to CQS strains indicate parasite-specific responses to CQ_GREEN_ exposure. The data contains valuable information when CQ_GREEN_ is used as an analogue for CQ.

## Background

*Plasmodium falciparum* is the main causative agent of malaria, killing hundreds of thousands of people annually [1]. Resistance to anti-malarial drugs is widespread, and promising anti-malarial drugs that are effective and affordable for people with low income are slow in development. For decades, chloroquine (CQ) was the safest, most affordable and effective drug against malaria, saving the lives of millions of people until resistance emerged [2]. To date, its mode of action is still not fully understood. Some researchers suggest that CQ could have more than one intracellular target, making it more difficult for parasites to develop resistance [3–5].

To effectively use CQ as an anti-malarial treatment in areas where CQ resistance has been reported, it is imperative to gain more insight into its mechanism of action. The small size and uncharged form of CQ at neutral pH makes it difficult to use for molecular biological experiments to determine its accumulation in intracellular compartments or its affinity to other molecules.

Chloroquine transport studies have mainly been performed using radiolabelled CQ [6–8]. The great advantage of this method is that the intrinsic properties of CQ remain unaltered. However, it is not possible to study intracellular distribution and trafficking of radiolabelled CQ in intact parasites. Therefore, fluorescently labelled CQ analogues provide a new means to evaluate intracellular activity of this drug. To date, there are two fluorescently labelled chloroquine analogues commercially available: LynxTag-CQ™_BLUE_ and LynxTag-CQ™_GREEN_ (BioLynx Technologies, Singapore, Singapore). While the literature cites that CQ_BLUE_ was mainly found to be fluorescent in the parasite cytosol [9–11], only one study has been published with CQ_GREEN_. This study showed CQ_GREEN_ accumulation in the DV of CQ-sensitive (CQS) *P. falciparum* parasites and yeast-derived microsomes expressing the *P. falciparum* chloroquine resistance transporter (PfCRT) [12].

For this study, CQ_GREEN_ was compared to unmodified CQ and investigated whether the fluorescently tagged CQ analogue can be used to obtain insight into intracellular CQ trafficking and localization. A better knowledge of the differences in CQ accumulation, distribution, uptake and efflux between CQS and CQ-resistant (CQR) strains is imperative to understand drug resistance. The findings show that, although CQ and CQ_GREEN_ show comparable IC_50_ values in CQS and CQR parasites, discrepancies were seen between CQ_GREEN_ and unmodified CQ in their expected intracellular localization. A strong CQ_GREEN_ fluorescence was mainly seen in the cytosol of both CQS and CQR strains.

## Methods

### Parasite strains and culture conditions

Two CQS (3D7, HB3) and two CQR (FCB, Dd2) strains were used for all experiments. Parasites were cultured continuously, as described by Trager and Jensen [13], with modifications. Briefly, parasites at 5% haematocrit were propagated in culture medium containing RPMI 1640 (Life Technologies, Burlington, ON, Canada) supplemented with 25 mM HEPES, 2 mM l-glutamine, gentamicin (20 µg/ml) (Life Technologies, Burlington, ON, Canada), 100 µM hypoxanthine (Sigma-Aldrich, Oakville, ON, Canada), and 0.5% AlbuMAX I (Life Technologies, Burlington, ON, Canada). Parasites were maintained at 37 °C with an atmosphere of 5% CO_2_, 3% O_2_ and 92% N_2_. A^+^ red blood cells were obtained from the Interstate Blood Bank (Memphis, TN, USA). Giemsa-stained blood smears were prepared daily to monitor parasite growth. For synchronization, parasites were treated with 5% d-sorbitol (BioShop Canada, Burlington, ON, Canada) for 10 min at 37 °C; sorbitol was removed and parasites were washed once before returning them back into culture.

### Cytotoxicity assays

Cytotoxicity assays were performed as described previously [14–17], with modifications. Cultures of 0.5% parasitaemia and 2% haematocrit were incubated in 100 µl culture medium per well in a 96-well plate assay. A drug dilution series of 1:3 was prepared, starting with 1 µM as highest substrate concentration. Plates were incubated at 37 °C, 5% CO_2_ and 3% O_2_ for 72 h, then frozen and stored at − 80 °C.

Readouts of the assay were performed using the SYBR Green I detection method. For this, plates were thawed at room temperature and 100 µl 2× lysis buffer (20 mM Tris pH 7.5, 5 mM EDTA, 0.008% saponin, 0.08% Triton X-100, and 0.2 µl SYBR Green I/ml) was added to each well. Plates were incubated in the dark for at least 1 h. Fluorescence intensity was determined using a Synergy H4 plate reader (Fisher Scientific, Nepean, ON, Canada) with 485 nm excitation and 520 nm emission wavelengths. IC_50_ values were determined by fitting concentration response curves with a custom-made procedure for IGOR Pro 6.2 based on a R script kindly provided by Le Nagard [18, 19].

### Fluorescence of CQ_GREEN_ at varying pH

To determine if fluorescence intensity of CQ_GREEN_ is altered at varying pH, buffer solutions were prepared ranging from pH 5.0–8.0 based on a modified Ringer’s solution (122.5 mM NaCl, 5.4 mM KCl, 1.2 mM CaCl_2_, 0.8 mM MgCl_2_, 11 mM d-glucose). Buffer solutions contained 10 mM MES for pH 5.0–6.5 or 10 mM HEPES for pH 7.0–8.0. In a 96-well plate, 100 µl buffer solutions of varying pH containing 1 µM CQ_GREEN_ were prepared in triplicate. Fluorescence intensity was measured at 37 °C using the Synergy H4 fluorimeter (Bio-Tek, Winooski, VT, USA). Excitation spectra ranging from 400 to 520 nm were measured with fixed emission at 540 nm, and emission spectra ranging from 500 to 630 nm were measured with fixed excitation at 488 nm. Quantification was done using Microsoft Excel 2013.

### Live cell imaging

Intracellular CQ_GREEN_ accumulation at different concentration and pH was analysed in intact parasitized red blood cells. For this, 3D7 trophozoite stage parasites were incubated for 30 min at 37 °C with 25, 50, 500 nM or 2.5 µM CQ_GREEN_ in Ringer’s solution with pH 7.4 or buffer solutions with pH 7.2 or 5.2. Images were taken using a 488 nm argon laser (12.5 mW, 0.8%) on a Zeiss LSM 710 confocal microscope (Carl Zeiss, Oberkochen, Germany) equipped with a water-corrected objective (C-apochromat 63×/1.20 W Korr M27). Emission range was set to 500–600 nm. Localization of CQ_GREEN_ within the parasite under various pH conditions was determined using the ZEN 2010 software (Carl Zeiss MicroImaging, Oberkochen, Germany).

For long-term incubation with CQ_GREEN_, early trophozoite stage 3D7 parasites were incubated with 100 nM, 300 nM and 500 nM CQ_GREEN_ for 7 h in culture medium at 37 °C, 3% O_2_, 5% CO_2_. Parasites were then transferred onto a microscope chamber and imaged using a Zeiss LSM 710 confocal microscope (Carl Zeiss, Oberkochen, Germany), a 63× water corrected objective (C-apochromat 63×/1.20 W Korr M27) and a 488 nm laser (12.5 mW, 0.8%). Emission range was set to 500–600 nm. A constant temperature of 37 °C was maintained during the measurements using a stage-top incubator (Tokai Hit, Shizuoka-ken, Japan). Images were analysed with the ZEN 2010 software (Carl Zeiss MicroImaging, Oberkochen, Germany).

To analyse CQ_GREEN_ uptake of CQS and CQR strains, synchronized trophozoite stage parasites were washed in Ringer’s solution and transferred onto a microscope chamber. Parasites were allowed to settle for 5 min, then the solution was aspirated and replaced with new Ringer’s solution containing 500 nM CQ_GREEN_. If verapamil was added, parasites were preincubated with 1 µM VP for 15 min at 37 °C, then transferred onto a microscope chamber. For the time lapse measurement, 500 nM CQ_GREEN_ were added to the Ringer’s solution with or without 1 µM VP. Images were taken every 3 s for a time span of 500 s using a Zeiss LSM 710 confocal microscope (Carl Zeiss, Oberkochen, Germany) and a 63× water corrected objective (C-apochromat 63×/1.20 W Korr M27). Excitation was done using a 488 nm laser (12.5 mW, 0.8%), and an emission range from 500 to 600 nm. Regions of interest (ROI) were set for the parasite cytosol, DV, infected RBC cytosol and uninfected RBC. Fluorescence of ROIs was determined for each time point using ImageJ 1.47q (National Institutes of Health, USA). Uptake rates were calculated for the cytosol and DV during the saturation phase and analysed using IGOR Pro 6.2 by fitting influx to ƒ = y_0_ + a(1 − e^−bx^) and initial rates (v_0_) to y = mx + b, as described previously [20]. Graphs were created using IGOR Pro 6.2.

## Results

### IC_50_ determination of CQ and CQ_GREEN_ in CQS and CQR parasite strains

To evaluate the efficacy of the fluorescently tagged chloroquine analogue CQ_GREEN_, the half maximal inhibitory concentrations (IC_50_) were determined using the SYBR Green I detection assay and a standardized calculation method for data analysis in two CQS and two CQR *P. falciparum* strains (Table 1) [19]. The two CQS strains 3D7 and HB3 had a CQ IC_50_ of 24 ± 6 nM and 14 ± 1 nM, respectively. Compared to exposure to CQ, IC_50_ values doubled in both CQ_GREEN_ treated 3D7 (48 ± 3 nM, p = 0.02) and in HB3 parasites (36 ± 8 nM, p = 0.06). Treatment of the CQR strains FCB and Dd2 showed similar IC_50_ values for all tested drugs. IC_50_ values of 166 ± 9 nM for CQ and 177 ± 43 nM for CQ_GREEN_ were determined in FCB (p = 0.81). For Dd2, IC_50_ values of 169 ± 4 nM were obtained for CQ and 174 ± 27 nM for CQ_GREEN_ (p = 0.85). Resistance in CQR strains could be reversed with the addition of 1 µM VP. For FCB, IC_50_ values were 41 ± 7 for CQ + VP and 30 ± 4 for CQ_GREEN_ + VP. For Dd2, IC_50_ values were 53 ± 7 for CQ + VP and 35 ± 6 for CQ_GREEN_ + VP. No significant changes were observed in CQS strains for treatment with CQ or CQ_GREEN_ in combination with verapamil.

**Table 1:** IC_50_ values of CQ and CQ_GREEN_ for *P. falciparum* strains used in this study

Strain	CQ	CQ + VP	CQ_GREEN_	CQ_GREEN_ + VP
3D7	24 ± 6	17 ± 1	48 ± 3	50 ± 3
HB3	14 ± 1	19 ± 2	36 ± 8	36 ± 7
FCB	166 ± 9	41 ± 7	177 ± 43	30 ± 4
Dd2	169 ± 4	53 ± 7	174 ± 27	35 ± 6

All values are given in nM ± SEM and represent three independent experiments with or without 1 µM VP

*VP* verapamil

### Fluorescence intensity of CQ_GREEN_ is dependent on pH

**Fig. 1:**
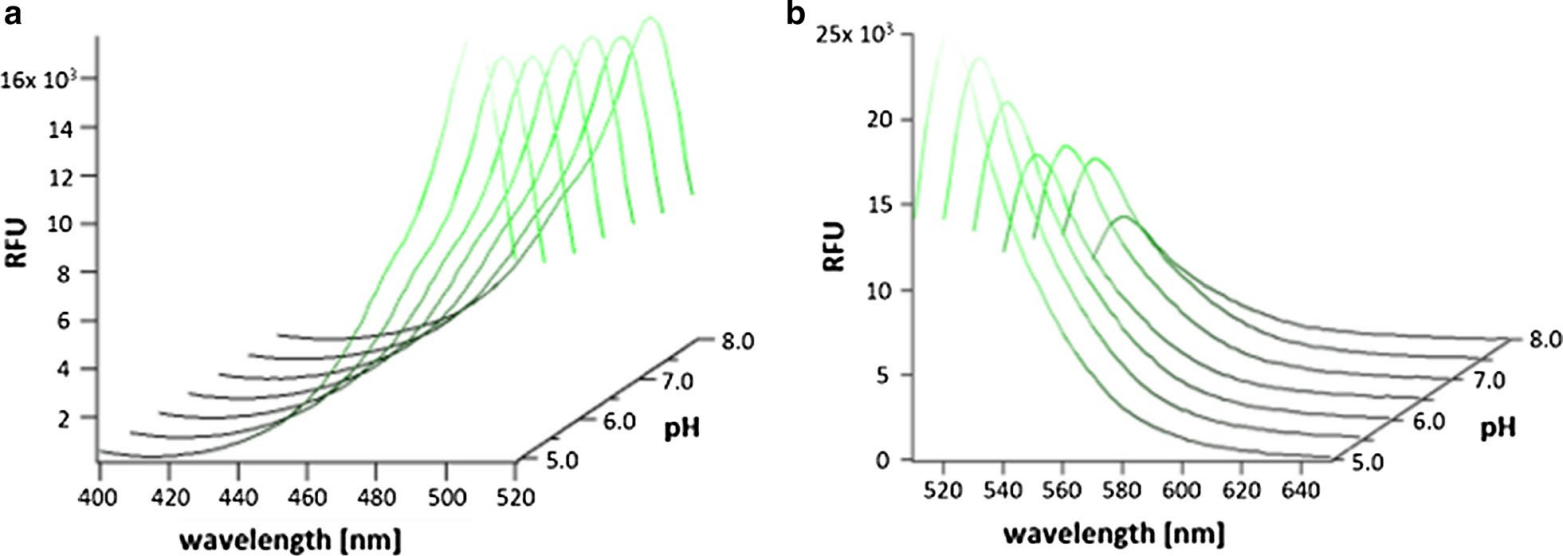
CQ_GREEN_ excitation and emission spectra at varying pH. Solutions with varying pH ranging from 5 to 8, each containing 1 µM CQ_GREEN_ were prepared in triplicate and measured using a fluorimeter. Temperature was set to 37 °C during measurements. **a** Excitation spectra with fixed emission at 540 ± 4.5 nm. **b** Emission spectra with fixed excitation at 488 ± 4.5 nm

pH is known to affect the fluorescence intensity and excitability of a molecule [21]. While some fluorophores have stable emission at different pH, others are more pH sensitive [22, 23]. This must be considered when comparing fluorescence intensity of a fluorochrome in intracellular compartments with significantly different pH, as is the case for the parasite’s cytosol (at approx. pH 7.1) and DV (at approx. pH 5.2) [24]. CQ_GREEN_ consists of a chloroquine analogue tagged with a Bodipy fluorophore, which is relatively insensitive to solvent polarity and pH [25]. To determine if this fluorescently tagged CQ is pH dependent, CQ_GREEN_ fluorescence was tested using buffer solutions containing HEPES or MES to obtain pH values ranging from 5.0 to 8.0. These buffer solutions were used to evaluate possible changes in fluorescence intensity seen in the parasite cytosol, having a physiological pH of 7.2, and the digestive vacuole, maintaining a pH of 5.2 [24]. Excitation spectra for CQ_GREEN_ showed constant fluorescence intensity at a fixed emission of 540 nm (Fig. 1a). The CQ_GREEN_ excitation peak was determined at 508 nm. Since live cell imaging will be performed using a 488 nm laser for excitation, CQ_GREEN_ emission spectra were measured at different pH with a fixed excitation wavelength of 488 nm. CQ_GREEN_ emission intensity peaked at 522 nm and increased 1.6-fold from 13,698 RFU at pH 7.5 to 22,419 RFU at pH 5.5 (Fig. 1b). Therefore, somewhat higher CQ_GREEN_ fluorescence is emitted in acidic compartments compared to neutral compartments at equal intracellular CQ_GREEN_ concentrations.

### CQ_GREEN_ accumulates in the parasite cytosol

**Fig. 2:**
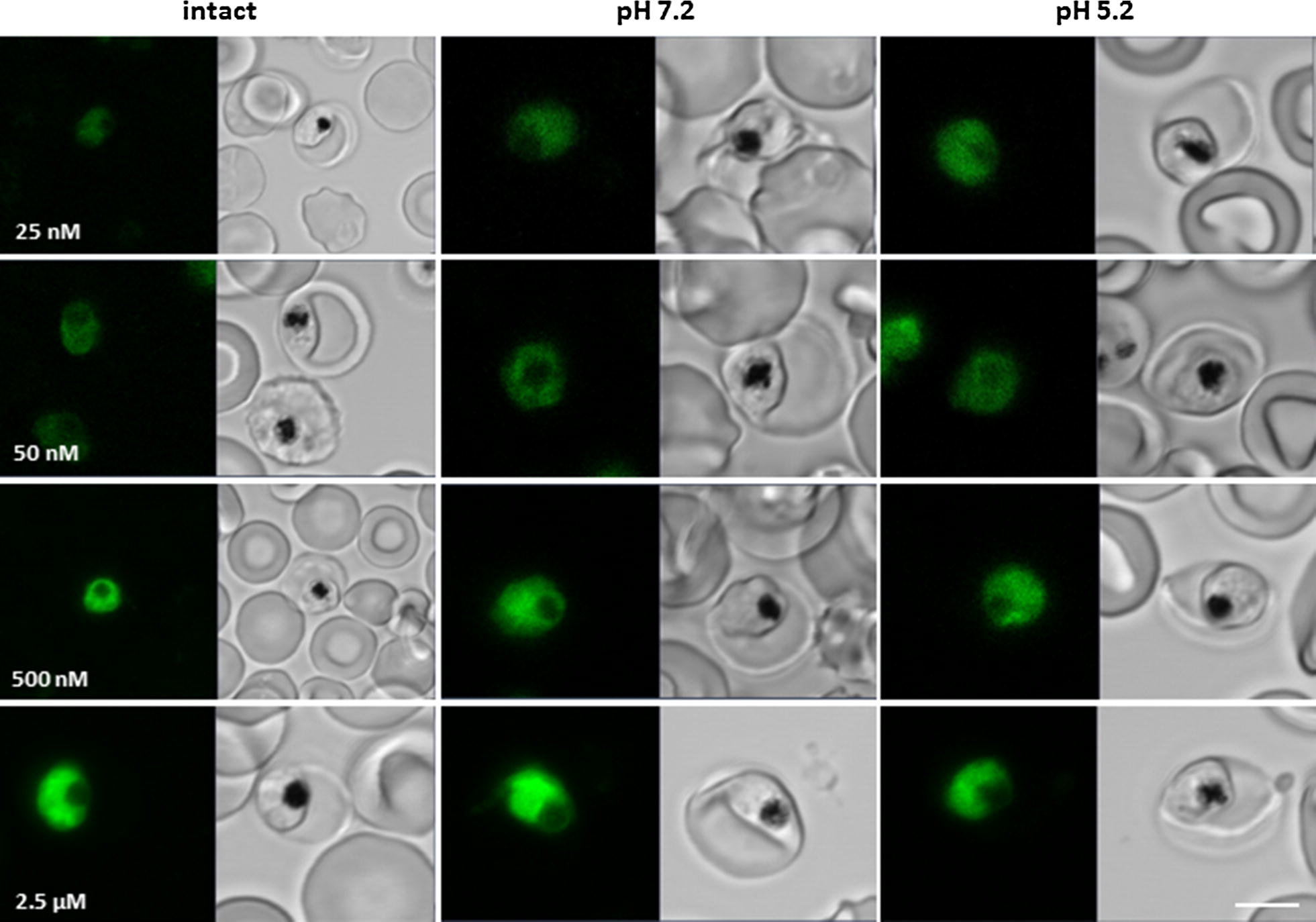
Live cell imaging of CQ_GREEN_ treated parasites. Synchronized trophozoite stage 3D7 parasites containing Ringer‘s or Equilibration Buffer solution for pH 7.2 and 5.2 were prepared for confocal microscopy. Different CQ_GREEN_ concentrations were added and uptake was monitored for 30 min, while emission range was set to 500–600 nm. Fluorescence increased with higher CQ_GREEN_ concentrations but no accumulation of CQ_GREEN_ could be detected in the parasite’s DV. Scale bar, 5 µm

The weak base properties of chloroquine and its diprotonation at low pH result in its high accumulation in the parasite’s digestive vacuole [26]. Addition of a fluorescent group (here Bodipy) to chloroquine could alter the intracellular distribution of this molecule. In this study, the CQS strain 3D7 was treated with 25 nM, 50 nM, 500 nM and 2.5 µM CQ_GREEN_ in Ringer’s solution, or buffer solutions with pH 7.2 and pH 5.2, respectively. CQ_GREEN_ uptake and intracellular distribution was monitored in 5 min intervals for a total of 30 min at 37 °C. Increasing CQ_GREEN_ concentrations resulted in stronger fluorescence signals, independent of the buffer solution used (Fig. 2). The fluorescence signal was always stronger in the cytosol compared to the DV. Faint accumulation of CQ_GREEN_ in the digestive vacuole was observed with all buffer solutions and CQ_GREEN_ concentrations.

**Fig. 3:**
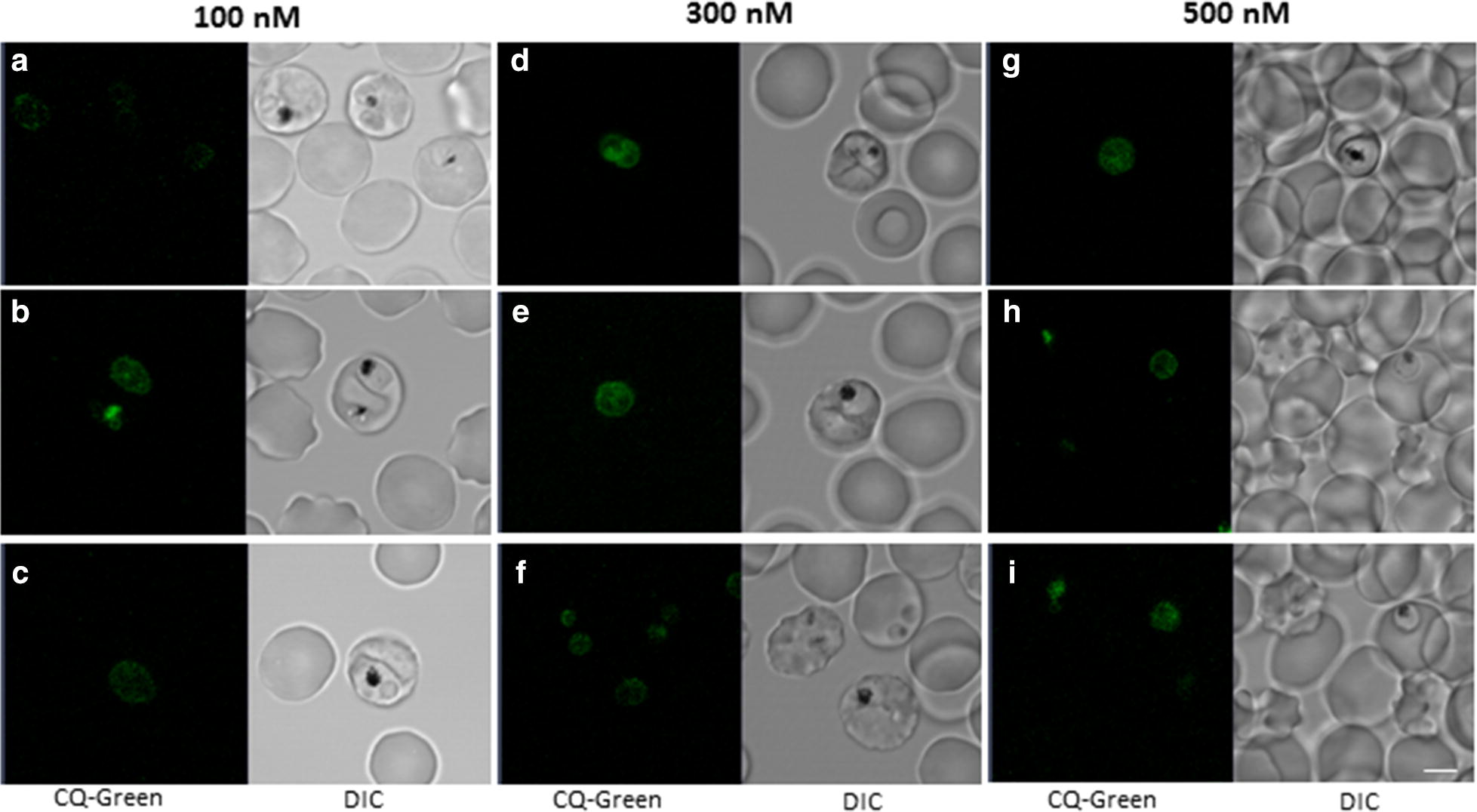
Long term incubation of CQ_GREEN_ treated parasites. Early trophozoite stage 3D7 parasites were incubated with 100 nM, 300 nM or 500 nM CQ_GREEN_ for 7 h in culture medium at 37 °C, 3% O_2_, 5% CO_2_, and then transferred onto a microscope chamber. Faint fluorescence accumulation of CQ_GREEN_ in the DV could be detected in few parasites (approx. 5%). Experiments were done in triplicate. **a–c** Images of parasites exposed to 100 nM CQ_GREEN_ for 7 h. **d–f** Images of parasites exposed to 300 nM CQ_GREEN_ for 7 h. **g–i** Images of parasites exposed to 500 nM CQ_GREEN_ for 7 h. Scale bar, 5 µm

Although CQ is expected to accumulate in the parasite’s DV within minutes, addition of CQ_GREEN_ was extended for up to 7 h in 3D7 parasites at 37 °C, 3% O_2_, 5% CO_2_, 92% N_2_. It was previously reported that incubation of the chloroquine analogue CQ_BLUE_ at a 3 µM concentration for 8 h resulted in nonspecific localization throughout the parasite’s cytosol, while accumulation of CQ_BLUE_ in the DV was detected using a concentration of 300 nM [9]. Therefore, for this study, CQ_GREEN_ concentrations ranging from 100 to 500 nM were used. Fluorescence signals obtained after 7 h incubation were low for all tested CQ_GREEN_ concentrations (Fig. 3). Accumulation of CQ_GREEN_ in the DV could be detected in very few parasites (approx. 5%) (Fig. 3b + d). Furthermore, accumulation of CQ_GREEN_ in the DV was only seen in one parasite of a double-infected red blood cell (RBC) (Fig. 3b), suggesting that CQ_GREEN_ accumulation in the DV is not equally achieved in individual parasites. Moreover, the CQ_GREEN_ fluorescence in the DV of the parasite in Fig. 3b is only 2.8-fold higher compared to the cytosol, which is much lower than expected.

### Stability of CQ_GREEN_ compound

Next, it was investigated if the Bodipy moiety of CQ_GREEN_ is cleaved and remains in the cytosol while CQ reaches the DV, where it can unfold its anti-malarial properties. It was previously demonstrated that cleavage of fluorescent molecules through proteases changes the environment of the reactive center loop, resulting in a spectral shift of the fluorescence peak [27, 28]. To evaluate if a spectral shift of the fluorescence peak occurs in CQ_GREEN_ after exposure to parasite proteases, uninfected or 3D7-infected cultures with a parasitaemia of 2.5%, 5% or 10% were loaded with 500 nM CQ_GREEN_ for 1 h. Parasites were then washed to remove excess CQ_GREEN_ in the supernatant and were lysed before measuring fluorescence. Since live cells were loaded with the dye, any detected signal should be derived from CQ_GREEN_ molecules taken up by the parasites. As a control, parasites were with free acid Bodipy-FL were analysed using the same conditions.

**Fig. 4:**
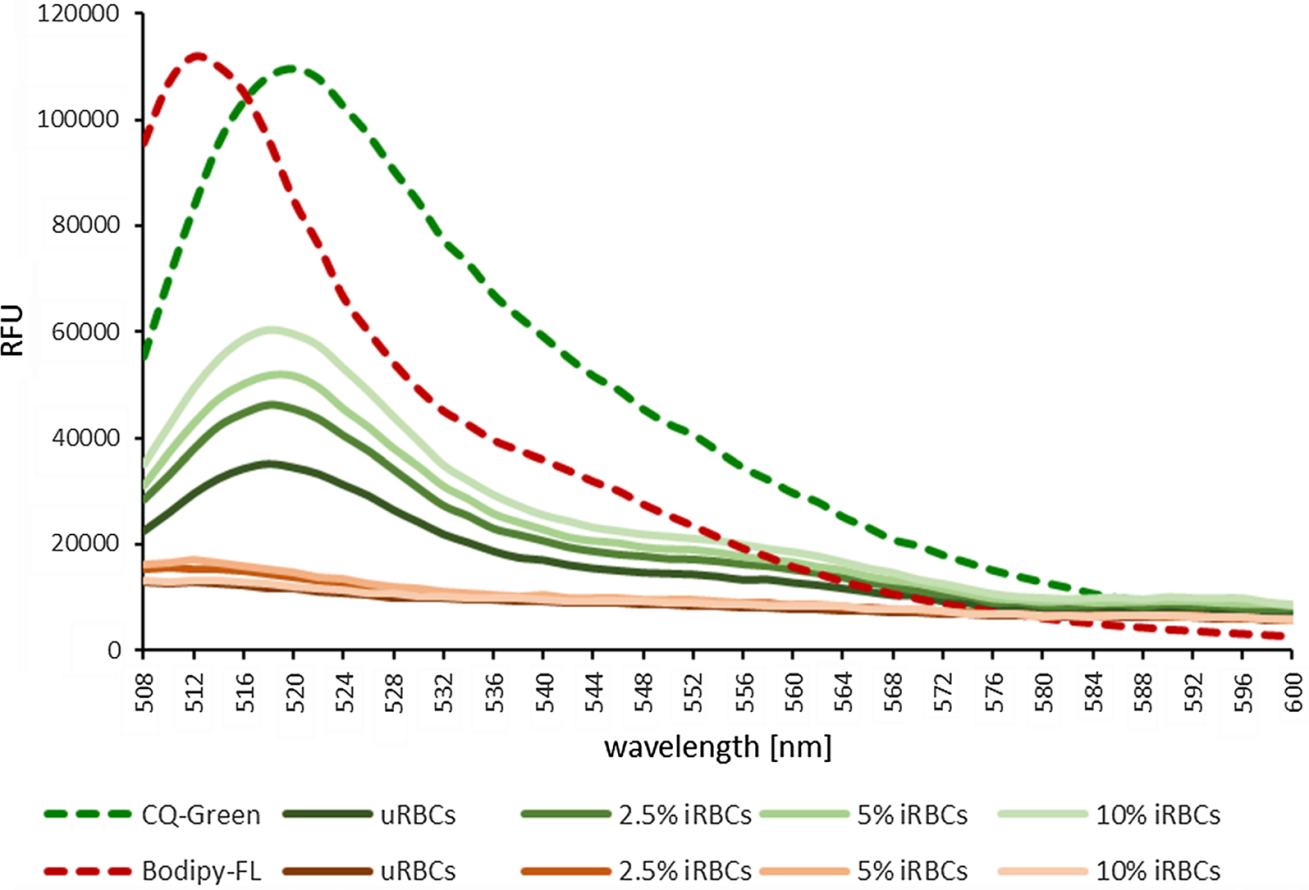
CQ_GREEN_ and Bodipy-FL fluorescence. CQ_GREEN_ and free acid Bodipy-FL were measured in solution or after incubation with, and lysis of, uninfected red blood cells and red blood cells infected with 3D7 trophozoite stage parasites (uRBCs and iRBCs, respectively). No shift of CQ_GREEN_ fluorescence towards the Bodipy-FL peak was observed in uRBCs and iRBCs. Experiment was done twice in triplicate

The fluorescence peak for the free acid Bodipy-FL control was measured at 512 nm, while the peak for CQ_GREEN_ was measured at 520 nm (Fig. 4). This fluorescence shift is large enough to differentiate between free Bodipy and Bodipy conjugated to CQ. Treatment of uninfected RBCs (uRBCs) and infected RBCs (iRBCs) with 500 nM Bodipy-FL for 1 h resulted in low fluorescence in all samples, indicating that Bodipy-FL is not readily accumulating in uRBCs or iRBCs. When parasites were treated with 500 nM CQ_GREEN_, a proportional increase in CQ_GREEN_ fluorescence was observed with increasing parasitaemia from 0% (uRBCs) to 10% (iRBCs), as expected. The fluorescence peak was measured at 520 nm for CQ_GREEN_ in Ringer’s solution alone (control) and remained constant at 518–520 nm for all CQ_GREEN_ treated uRBC and iRBC samples. Thus, either no cleavage of the Bodipy tag in CQ_GREEN_ occurred or the cleaved Bodipy moiety was not retained within the RBCs and a fluorescent signal was only obtained from uncleaved CQ_GREEN_.

### CQ_GREEN_ uptake differs in CQS and CQR strains

Most researchers focus on CQ accumulation in the DV, suggesting that enhanced CQ efflux from the DV through PfCRT confers drug resistance [29, 30]. CQ uptake in intact iRBCs has mainly been described as passive diffusion and subsequent accumulation in the DV due to the drug’s weak base properties [31, 32]. Molecular mechanisms that influence CQ uptake and, therefore, reduce the drug influx in CQR strains compared to CQS strains may have gone unrecognized. CQ_GREEN_ offers the possibility to measure uptake in live cells. Although CQ_GREEN_ mainly accumulates and fluoresces in the parasite’s cytosol, with weak fluorescence in the DV, it was still possible to measure its uptake rate in both compartments.

**Fig. 5:**
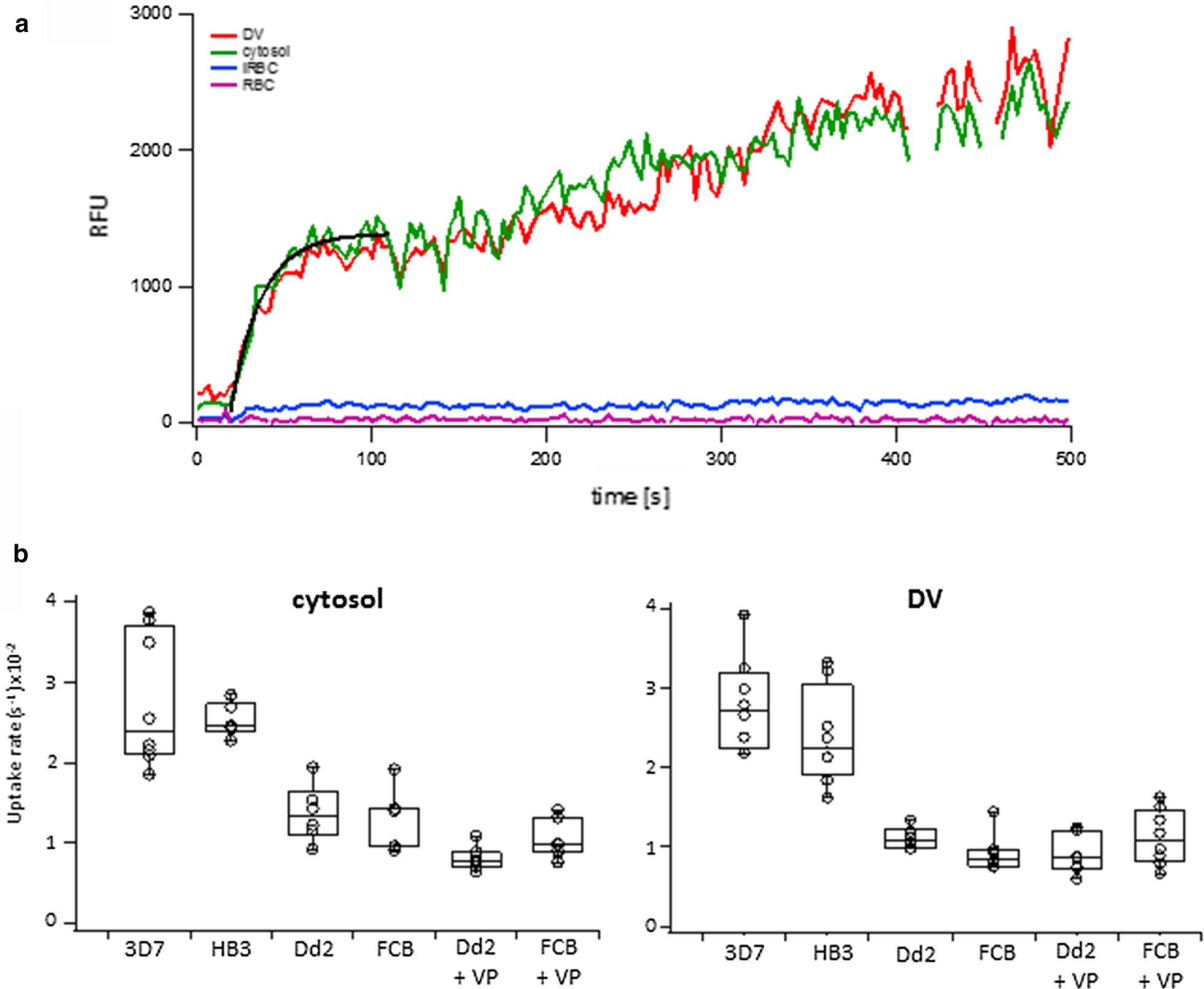
CQ_GREEN_ uptake in CQS and CQR parasites. Trophozoite stage parasites were incubated with CQ_GREEN_ and fluorescence was recorded every 3 s. **a** Fluorescence was measured in the parasite cytosol, DV, infected RBC (iRBC) cytoplasm and uninfected RBC (uRBC). No accumulation of CQ_GREEN_ was observed for the uRBC and iRBC cytoplasm. Uptake rates were calculated for the parasite cytosol and DV during the saturation phase (black curve), which was followed by a linear uptake phase. **b** CQR strains showed a slower CQ_GREEN_ uptake rate compared to CQS strains in both the cytosol and the DV of the parasite. Addition of verapamil (VP) did not influence the CQ_GREEN_ uptake rate in CQR strains. Experiments were done in triplicate

The rate of CQ_GREEN_ uptake was analysed in two CQS strains (3D7 and HB3) and compared with two CQR strains (Dd2 and FCB). Fluorescence was measured from the parasite cytosol, DV, iRBC cytoplasm and uRBC. No increase in CQ_GREEN_ fluorescence beyond background levels was observed in the cytoplasm of infected or uninfected RBCs (Fig. 5a). There was a rapid increase in fluorescence in the parasite cytosol and DV in the initial 150 s, followed by a slower, almost linear CQ_GREEN_ uptake. This is in agreement with previous studies where they showed that CQ uptake can be separated into a short period of very rapid uptake (< 30 s) followed by a long, slower phase [33], and addition of verapamil did not influence CQ uptake during the initial phase [34].

CQ_GREEN_ uptake rate for the parasite cytosol was approx. twofold higher in CQS strains compared to CQR strains (Fig. 5b). For the DV, CQ_GREEN_ uptake rates were approx. 2.5-fold higher in CQS strains compared to CQR strains. CQ_GREEN_ uptake rates between CQS and CQR strains were statistically highly significant for both the cytosol (p < 0.0001) and DV (p < 0.0001). Increased CQ_GREEN_ uptake rates for the DV of CQS strains suggest that active transport of CQ into the DV occurred in addition to diffusion, which is absent in CQR strains. As expected, addition of verapamil did not influence CQ_GREEN_ uptake in the parasite cytosol or DV in all CQR strains tested.

## Discussion

The exact mechanisms responsible for chloroquine resistance have eluded researchers for decades. It has long been described that CQR strains accumulate two to sevenfold less CQ than CQS strains [35]. Nevertheless, CQR strains are still able to tolerate higher intracellular CQ concentrations than CQS strains before irreversible cell damage occurs [3, 7, 33, 36]. Researchers have advocated that inhibition of haemozoin formation by CQ is not sufficient to explain its effect on parasite killing [37]. Furthermore, a possible role of CQ in the parasite’s cytosol has also been proposed [5]. A fluorescently labelled CQ analogue could provide insight into the intracellular distribution of CQ that is not attributed to diffusion alone. This study set out to examine CQ uptake in live parasites using CQ_GREEN_.

IC_50_ values were compared in CQS and CQR strains after exposure to CQ or CQ_GREEN_. A decrease in the efficacy for CQ_GREEN_ may suggest that the CQ analogue does not reach its site of action as efficiently as its native form or binds to its target less efficiently. Calculated IC_50_ values in CQS strains showed that CQ was twice as effective as CQ_GREEN_ in parasite growth inhibition. In comparison, similar IC_50_ values for CQ and CQ_GREEN_ were determined in CQR strains. In a previous study by Loh and colleagues [12], IC_50_ values in 3D7 were approx. fivefold higher for CQ_GREEN_ compared to CQ, and nearly doubled in the CQR strain K1. Thus, the parasites used for this study seemed to be more sensitive to CQ_GREEN_ exposure than reported in earlier publications. In both studies, drug resistance could be reversed by chemosensitizers such as verapamil, suggesting that CQ_GREEN_’s mode of action is similar to CQ.

Although CQ_GREEN_ seemed slightly less effective than CQ in the IC_50_ assays for CQS strains, its Bodipy fluorescent tag makes it suitable for live cell imaging. This was confirmed by fluorometric readings, where a strong fluorescence emission signal for CQ_GREEN_ was measured at a 488 nm excitation wavelength. Spectral scans showed a strong fluorescence signal at the measured pH spectrum ranging from pH 5–8 with a moderate increase in fluorescence at acidic pH.

Accumulation of CQ_GREEN_ fluorescence was found in only 5% of the parasite’s DV compared to the cytosol during live cell imaging at any of the tested CQ_GREEN_ concentrations, ranging from 25 nM to 2.5 µM. Considering that the CQ_GREEN_ IC_50_ was determined at 24 nM, any of the tested concentrations for live cell imaging would have been sufficient to kill the parasites. If CQ and CQ_GREEN_ had its primary target in the parasite’s DV, as suggested by several studies [38–42], then we would have expected a higher CQ_GREEN_ fluorescence in the DV.

Protonation of CQ, or its analogues, influence their membrane permeability and thus their intracellular distribution [43, 44]. Treatment of intact *P. falciparum*-infected erythrocytes with pH buffered solutions ranging from pH 5–8 showed that protonation did not play a role in the intraparasitic CQ_GREEN_ distribution. One study used microsomes to resemble events occurring in the DV and reported accumulation of CQ_GREEN_ fluorescence in these microsomes [12]. They did not use intact parasites in their experiments and the data does not support their assumption that CQ_GREEN_ accumulates equally well in the DV of live parasites as it does in microsomes [12].

Fluorometric measurements were performed to verify CQ_GREEN_ fluorescence at low pH. Slightly higher fluorescence was observed for CQ_GREEN_ at acidic pH (5.0) compared to neutral pH (7.0). Therefore, if CQ_GREEN_ accumulated in the DV, a fluorescent signal would be expected. The absence of fluorescence in the DV suggests that CQ_GREEN_ does not reach the DV. Two reasons for this are possible. First, cleavage of the fluorescent Bodipy moiety from CQ may already occur in the parasite cytosol. This would allow CQ to accumulate in the DV, while the Bodipy moiety remains in the cytosol. In this case, the fluorescence signal does not specify the subcellular localization of CQ but rather only the fluorochrome. Second, the Bodipy moiety alters the intrinsic properties of the substrate and, therefore, prevents its accumulation in the DV. A simple way to elucidate whether the Bodipy moiety gets cleaved is determining fluorescence properties of conjugated versus free acid Bodipy-FL. Since there is a fluorescence shift between free acid Bodipy-FL alone (peak at 512 nm) and the Bodipy-tagged CQ_GREEN_ (peak at 520 nm), cleavage of the Bodipy moiety from CQ_GREEN_ during the cellular uptake would result in a fluorescence shift to 512 nm when parasites are treated with CQ_GREEN_. For this study, intact *P. falciparum*-infected RBCs were treated with CQ_GREEN_ to analyse the potential cleavage of the Bodipy moiety. Although a proportional increase in fluorescence with higher parasitaemia was measured, no shift in the fluorescence peak was observed. Thus, CQ_GREEN_ remained intact and functional in live parasites prior to parasite lysis for fluorescence measurements.

Despite the unexpected localization of CQ_GREEN_ fluorescence mainly in the parasite cytosol and to a lesser extent in the DV, uptake rates were analysed in two CQS and two CQR strains. Although the fluorescent signal obtained from the DV was weak compared to the cytosol, it was sufficient to calculate the uptake rate for this compartment. CQS strains had approx. twofold higher CQ_GREEN_ uptake rates for the cytosol and 2.5-fold higher rates for the DV compared to CQR strains. Addition of verapamil to the CQR strains did not alter CQ_GREEN_ uptake rates, suggesting that PfCRT does not play a role in its uptake. This is consistent with the hypothesis that PfCRT is involved in CQ efflux from the DV and is not involved in its uptake [20]. Decreased CQ uptake rates in CQR strains compared to CQS strains may be explained through reduced availability or affinity to an intracellular target, such as free haem [4]. Thus, accumulation of CQ in the DV may contribute to parasite killing, but additional drug targets in the parasite cytosol could also play a role.

## Conclusion

Live cell imaging using fluorescently tagged anti-malarial drugs provides a great tool to elucidate their intracellular localization and give insights into their uptake or efflux rates. The challenge using fluorescently labelled CQ analogues is their sensitivity to pH and altered intracellular localization, compared to native CQ. Finding fluorescent groups that are more stable at different pH and do not influence the diffusion properties of the protein would provide a powerful tool in studying the activity of anti-malarial drugs. Alterations between drug-sensitive and -resistance strains can be closely monitored to enhance our understanding of resistance mechanisms. Moreover, affinity of CQ, or its analogues, to cytosolic proteins may direct research on new anti-malarial drug design away from the DV and increase the focus on cellular pathways in the parasite cytosol.

## Data Availability

The data are available on request from the authors.

## Abbreviations

CQ: chloroquine
CQ_GREEN_: chloroquine analogue LynxTag-CQ™_GREEN_
CQR: chloroquine resistant
CQS: chloroquine sensitive
DV: digestive vacuole
IC_50_: half maximal inhibitory concentration
PfCRT: *Plasmodium falciparum* chloroquine resistance transporter
RBC: red blood cell
iRBC: *Plasmodium falciparum* infected red blood cell
uRBC: uninfected red blood cell
